# POEMS Syndrome: A Report of 14 Cases and Review of the Literature

**DOI:** 10.5402/2012/584287

**Published:** 2012-04-01

**Authors:** Zong Fei Ji, Dan Ying Zhang, Shu Qiang Weng, Xi Zhong Shen, Hou Yu Liu, Ling Dong

**Affiliations:** Department of Gastroenterology, Zhongshan Hospital, Shanghai Medical College, Fudan University, Shanghai 200032, China

## Abstract

POEMS syndrome is a rare paraneoplastic disorder associated with an underlying plasma cell dyscrasia presenting polyneuropathy, organomegaly, endocrinopathy, monoclonal protein, and skin changes. This study reviewed the clinical characteristics of 14 POEMS patients in Zhongshan hospital. The ratio of male to female was 9 : 5, and the average age was 47.1 years. The clinical manifestations were various, including motorial symptoms (weakness), sensory symptoms (numbness), lymphadenopathy, edema, abdominal distention, and skin hyperpigmentation. Imaging studies and laboratory tests also exhibited hepatomegaly, splenomegaly, thrombocytosis, endocrinopathy, and positive serum immunofixation in most patients. In addition, increased plasma cells in bone marrow and Castleman Disease were found in bone marrow and lymph nodes biopsies. All the eight follow-up patients were treated with alkylator-based combination chemotherapy or corticosteroids and thalidomide, with or without autologous stem cell transplantation. Unfortunately, two patients died three or four years after diagnosis of POEMS syndrome. The others showed response to therapy to some extent, but not completely remission. Currently, treatments for POEMS include radiation to the plasmacytoma, and systemic therapy is indicated. Low-dose alkylators with or without corticosteroids are effective in some patients. However, high-dose chemotherapy with auto-SCT dramatically improved symptoms and outcomes for POEMS patients.

## 1. Introduction

POEMS syndrome is a rare paraneoplastic disorder associated with an underlying plasma cell dyscrasia [1]. The acronym POEMS refers to frequently occurring features of the syndrome, including polyneuropathy, organomegaly, endocrinopathy, monoclonal protein (M-protein), and skin changes [1–5]. It was also called osteosclerotic myeloma, Crow-Fukase syndrome, and Takatsuki syndrome [3, 6] and was first reported by Scheinker in 1938 [3, 7]. In 1986, the first Chinese case of POEMS syndrome had been described [8]. Here, we report 14 clinical cases of POEMS syndrome in Zhongshan Hospital, Fudan University, and review of the literature. 

## 2. Patients and Methods

From 2005 to 2010, 14 POEMS patients were admitted to our hospital. All 14 patients met the diagnosis criteria proposed by Nakanishi et al. in 1984 [3] or Dispenzieri et al. in 2003 [9]. Nankanish et al. in 1984 first proposed the criteria of POEMS syndrome containing polyneuropathy, organomegaly (hepatomegaly, splenomegaly, or lymphadenopathy), endocrinopathy (hypothyroidism, diabetes mellitus, hypoadrenocorticism, or hypogonadism), M-protein, skin changes (hyperpigmentation, hypertrichosis, or thickening), and effusion or peripheral edema. At least three of these six criteria are required for diagnosis. Dispenzieri et al. [9] in 2003 suggested the criteria for the diagnosis of POEMS syndrome. The major criteria included polyneuropathy and monoclonal plasmaproliferative disorder. The minor criteria included sclerotic bone lesions, Castleman disease, organomegaly, edema, endocrinopathy, skin changes, and papilledema. Two major criteria and at least 1 minor criterion are required to diagnose POEMS syndrome.

We analyzed their general information, symptoms and signs, laboratory tests, therapy, and survival. Eight of the 14 patients got followed up in the clinic or by telephone for survival. The last follow-up day was August 4, 2011.

## 3. Result

### 3.1. General Conditions and Clinical Manifestations

From 2005 to 2010, 14 patients were diagnosed of POEMS syndrome in our hospital. The ratio of male to female was 9 : 5, and the average age was 47.1 years (range: 39–68 years). The initial clinical manifestation in this study was multifarious, including motorial symptoms (weakness) (57.1%), sensory symptoms (numbness) (35.7%), skin hyperpigmentation (78.6%), edema (50%), and abdominal distention (37.3%). The typical clinical features of these patients were listed in Table 1.

#### 3.1.1. Polyneuropathy

All the patients had peripheral neuropathy confirmed by nerve conduction/EMG studies. Among these patients, eight had motorial deficit (57.1%), while five had sensory deficit (35.7%). Meanwhile, five patients (35.7%) presented sensorimotor deficit.

#### 3.1.2. Organomegaly

 Organomegaly was the universal signs of all the patients (92.9%), in which splenomegaly (84.6%) was more common than hepatomegaly (61.5%) and lymphadenopathy (50.0%). Multiorganomegaly could be seen in 76.9% of them.

#### 3.1.3. Endocrinopathy

 Among the 14 patients of POEMS syndrome, 10 patients suffering from endocrinopathy all presented as hypothyroidism. In these patients, one had amenorrhea, two had impotence, one had hypoadrenocorticism, and one had hypoparathyroidism.

#### 3.1.4. Monoclonal Plasmaproliferative Disorder

Of the 13 patients who had serum immunofixation detection, ten (76.9%) showed positive results of monoclonal plasma proliferative disorder. Six patients had abnormal immunoglobulin A (IgA) *λ*, two had IgG *λ*, one had IgA *κ*, and the rest had IgM *λ*. Another patient had both serum IgG and IgA elevation, while serum immunofixation remained negative.

#### 3.1.5. Skin Changes

 Of all the 14 patients, the most common abnormality was hyperpigmentation (11 patients, 78.6%), followed by pruritus (two patients, 14.2%).

#### 3.1.6. Edema and Effusions

 In our study, most of the patients had edema and effusions of varying degrees. Seven had peripheral edema (50%), and 11 had serouscavity effusions (78.6%) including ascites (71.4%), pleural effusion (57.1%), hydropericardium (57.1%). Multiple serous cavity effusions were present in nine patients (64.3%).

#### 3.1.7. Papilledema

 Ten patients underwent fundus examination, and five were diagnosed with papilledema.

### 3.2. Accessory Examination

Blood routine and urinalysis of the 14 patients revealed that five of them had thrombocytosis (35.7%), two had microscopic haematuria (14.3%), and four had proteinuria (28.6%). Renal insufficiency occurred in five patients (35.7%). One of them experienced renal biopsy and was diagnosed with IgA nephropathy.

Laboratory tests exhibited that 10 patients had thyroid hypofunction. Hypoadrenocorticism and hypoparathyroidism were seen in individual patient. The concentration of gonadal hormone lowered significantly in two patients (1 male and 1 female).

 Three of the seven patients who had lymphadenopathy underwent lymph node biopsy. All were diagnosed with Castleman disease histopathologically. One sample showed *λ*-light chain (+) after immunohistochemical staining indicating monoclonal plasmaproliferative disorder.

Nerve conduction/EMG studies of all patients showed prolonged distal motorial latency and slowed velocity of both motorial and sensory nerve conduction. One patient had sural nerve biopsy and showed infiltration of lymphocytes around vessels in epineurium and partial demyelination while neuraxon intact.

Among the 14 patients, 11 had bone marrow aspiration. Two patients (18.2%) had a slight increase in plasma cells (more than 2%). Two (18.2%) had more than 5% plasma cells, but no definite signs of multiple myeloma. The rest had normal plasma cells. In patients who had radiographic bone survey and isotope bone scan, no sclerotic or lytic lesions were found.

Three of the eight follow-up patients were treated with alkylator-based combination chemotherapy. Five patients were treated with corticosteroids and thalidomide, and one also had autologous stem cell transplantation (auto-SCT). Two patients treated by alkylator-based combination chemotherapy died three or four years after the diagnosis of POEMS syndrome. All the others were responsive to therapy to some extent but not completely relieved.

## 4. Discussion

POEMS syndrome was first reported by Scheinker in 1938 [3, 7]. It is a multisystem disorder associated with polyneuropathy, organomegaly, endocrinopathy, M-protein, and skin changes [10, 11]. Elevation of proangiogenic and pro-inflammatory cytokines are the hallmark in the pathogenesis of this disorder [12–15]. High levels of interlcukin-1*β* (IL-1*β*), interlcukin-6 (IL-6), tumor necrosis factor-*α* (TNF-*α*), and vascular endothelial growth factor (VEGF) were detected in the serum of POEMS patients [12–15]. Among these cytokines, VEGF, probably oversecreted by plasma cells, appears to be the dominant driving cytokine and may be causative for effusions, pulmonary hypertension, and DIC in POEMS syndrome [16, 17].

Dispenzieri et al. investigated 99 patients in 2003. The median age and the ratio of men to female were similar to that of our study. The major clinical feature of this syndrome is a chronic progressive polyneuropathy with a predominant motorial disability [1]. In our study, the initial symptoms of most patients were sensory and also motorial symptoms (Table 1). However, all of the 14 patients were diagnosed with polyneuropathy by nerve conduction/EMG studies even including those who did not complain about numbness or dysesthesias. The neuropathy is usually symmetrical and ascending, with either insidious or rapidly progressing onset [9], and finally POEMS patients may be confined to a wheelchair.

Organomegaly usually affects the liver, the spleen, and the lymph nodes. Among our patients, the splenomegaly was the most common symptom (84.6%), which mirrors to the report of Cui et al. [10]. Nevertheless, Li et al. [18] reviewed the clinical characteristics of 99 Chinese POEMS patients and found 74 patients with lymphadenopathy, 70 with splenomegaly, and 47 with hepatomegaly. Dispenzieri et al. described that the liver was palpable in almost one-half of patients, but splenomegaly and lymphadenopathy were found in fewer patients [1].

The M-protein is typically small (median 1.1 g/dL, rarely more than 3.0 g/dL) and remains undetected on serum-protein electrophoresis without immunofixation. The M-protein is usually IgG or IgA and almost of the lambda type [1, 10]. According to Dispenzieri et al. [9], all patients had evidence of a monoclonal plasmaproliferative disorder, and 87% had a detectable M-protein in their serum or urine by immunofixation. For the 12 patients whose serum or urine M-protein remained undetected, the expression of M-protein was demonstrated by immunohistochemical staining of biopsy specimens from sclerotic bone lesions or bone marrow. In our study, 10 out of 13 patients (76.9%) had a visible M-protein by immunofixation. The incidence of those was significantly lower than reported by Dispenzieri et al. [9]. Among these 10 patients, one patient was further verified *λ* (+) by immunohistochemical stain of his lymphonode biopsy specimen. We should find more positive M-protein in other patients if we could check the serum or urine immunofixation repeatedly or proceed with immunohistochemical stain on biopsy samples of lympho node, sclerotic bone lesions, or bone marrow.

Our research exhibited that 14.3% and 28.6% of the patients had hematuria and proteinuria. Elevated Cr level in serum was detected in 35.7% of our patients, indicating that POEMS may lead to renal impairment or even renal failure. The result was similar to the report of Li et al. [18]. The histopathologic alteration of glomerulus usually present as membranoproliferative glomerulonephritis (MPGN) [19]. Based on the impacts of IL1-*β* and IL-6 as the mediators of endothelial cell activation, especially VEGF as an endothelial cell mitogen and an inducer of microvascular permeability, an endothelial cell-mediated injury is more likely to be implicated in the pathogenesis of this syndrome [14, 18, 20].

In our study, majority of our patients did not have radiographic bone survey owing to our tight turnover rate. According to Dispenzieri et al. [9], 97% of their patients had at least one abnormality detected on bone radiography. About half of patients had mixed sclerotic and lytic lesions.

Apart from the above, the important traits of POEMS syndrome include elevated levels of VEGF, sclerotic bone lesions, Castleman disease, papilledema, peripheral edema, ascites, effusions, thrombocytosis, and polycythemia [1]. In addition, we found that Castleman disease, papilledema, peripheral edema, ascites, effusions, and thrombocytosis were also common manifestations.

Based on the earlier criteria in 2003 [9], Dispenzieri [1] revised the diagnostic criteria of POEMS syndrome in 2007. He proposed that sclerotic bone lesions, Castleman disease, and elevated levels of VEGF as major diagnostic criteria. Apart from polyneuropathy and monoclonal plasma cell disorder, in order to make diagnosis at least one other major criterion and 1 minor criterion are required.

Currently, the treatments for POEMS syndrome include radiation, alkylators, corticosteroids, auto-SCT, bevacizumab, rituximab, bortezomib, and thalidomide [2, 21–30]. In patients with a dominant sclerotic plasmacytoma, first-line therapy should contain radiation therapy to the lesion. For those patients with systemic manifestation, systemic therapy is indicated. Low dose alkylators with or without corticosteroids are effective in some patients. Furthermore, high-dose chemotherapy with auto-SCT dramatically improved manifestations and outcomes for POEMS patients [2, 21, 24, 31].

In 2009, Ohwada et al. [29] reported the first case of successful combination induction therapy of bevacizumab (anti-VEGF MoAb) and thalidomide, followed by Auto-SCT. Bevacizumab could induce a dramatic decrease in the serum VEGF level, and thereby reduce pleural effusion and ascites with thalidomide to maintain low VEGF level. In summary, bevacizumab is efficacious for POEMS syndrome, when it is used in combination with other chemotherapeutics at the earlier stage.

Another successful case of POEMS syndrome associated with Waldenstrom macroglobulinemia (WM) was treated by rituximab with thalidomide [26], which effectively decreased CD20-positive lymphoplasmacytic cells and improved patient's neurological symptoms.

The clinical course of POEMS syndrome is chronic. A 93-patient follow-up data revealed that the median survival of patients with POEMS syndrome is 165 months [9]. Another research in China showed 80% patients were alive after follow-up time of 25 months, and 10% patients had survived more than 60 months [18]. The neuropathy, along with stroke and myocardial infarction, is the cause of death at last [1, 9].

## Figures and Tables

**Table 1:** Clinical features of 14 patients.

Polyneuropathy	
Peripheral neuropathy	14/14
Papilledema	5/10

Organomegaly	
Hepatomegaly	8/14
Splenomegaly	11/14
Lymphadenopathy	7/14

Endocrinopathy	
Hypothyroidism	10/14
Amenorrhea	1/14
Impotence	2/14
Hypoadrenocorticism	1/14
Hypoparathyroidism	1/14

M-protein	
IgA *λ*	6/13
IgA *κ*	1/13
IgM *λ*	1/13
IgG *λ*	2/13

Skin change	
Hyperpigmentation	11/14
Pruritus	2/14

Edema	
Peripheral edema	7/14

Effusions	10/14
Ascites	
Pleural effusion	8/14
Hydropericardium	8/14
